# Sports-related concussion (SRC) in track cycling: SRC assessment protocol for elite track cycling

**DOI:** 10.1136/bmjsem-2022-001384

**Published:** 2022-08-16

**Authors:** Clint Gomes, Nigel Jones, Neil Heron

**Affiliations:** 1 Department of Medicine, English Institute of Sport, Manchester, UK; 2 Department of Medicine, British Cycling, Manchester, UK; 3 General Practice/Centre for Public Health, Queen's University Belfast, Belfast, UK; 4 School of Medicine, Keele University, Keele, England

**Keywords:** concussion, cycling, trauma

## Abstract

Track cycling is a fast, exciting sport and requires a specific sports-related concussion (SRC) assessment protocol. This paper proposes the first SRC assessment protocol for use in track cycling and proposes that this should occur in three stages. Stage 1 will occur at the trackside, whilst stage 2 occurs in the changing room immediately after the event and stage 3 the day following the suspected SRC. This SRC protocol is in its first iteration and we hope it stimulates debate to allow further refinement.

**Key messages**

- Sports related concussion (SRC) is common within track cycling.
- This paper presents a protocol for assessing, diagnosing and managing SRC in track cycling.
- This proposed SRC assessment protocol is the first step in the process and we hope it stimulates debate within the cycling and sport medicine community.

## Track cycling

Track cycling is an exciting sport comprising both sprint and endurance events. Sprinters can reach speeds over 80 km per hour while endurance events involve up to 200 laps of a velodrome track. All cycling disciplines risk crashing; unfortunately, recent examples exist where cyclists have returned to the track despite being concussed.^1^,^2^ Track cycling does not currently have a specific sports-related concussion (SRC) assessment protocol,^3^ including a trackside screening (‘go/no go’) assessment protocol. Therefore, we would like to propose the creation of a track cycling-specific SRC assessment protocol for use trackside, in keeping with other cycling disciplines.^4^

## The solution: track cycling SRC assessment protocol

To account for the often transient, evolving or delayed onset of SRC symptoms, serial clinical evaluations should be embedded within a three-stage process to optimise SRC diagnosis.^5^ This track cycling SRC assessment will be modified as epidemiological data on SRC in track cycling becomes available, and comments are received from those involved in the sport and in wider SRC policy development.

**Figure 1:**
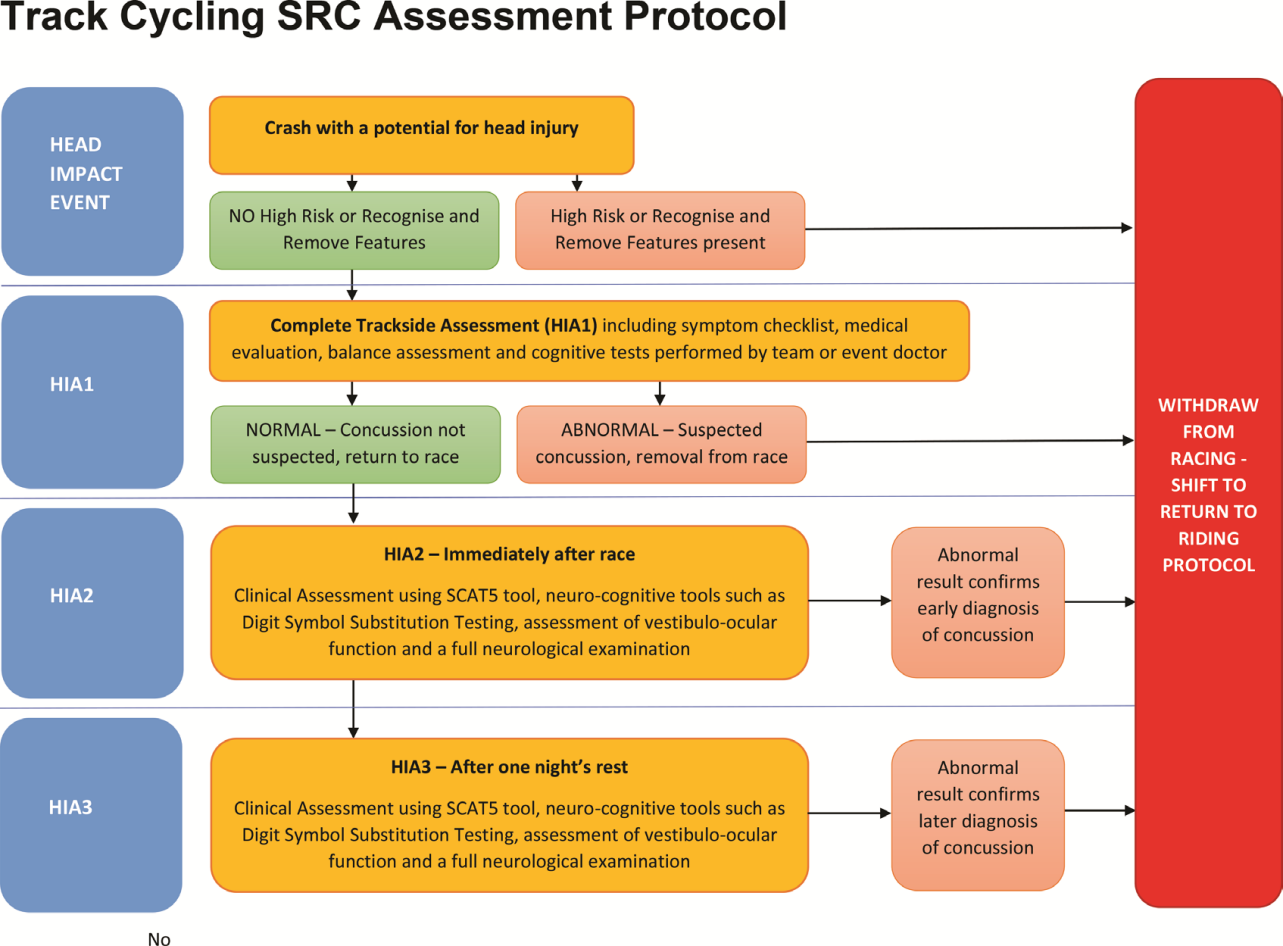
Initial head injury assessment. LOC, loss of concussion.

Those suspected of having an SRC event should be assessed in three stages (see figure 1), similar to other sports.^6^

1. Immediate trackside testing following a suspected SRC event (HIA1).

2. Retesting after completion of the track event on the same day of the suspected SRC event (HIA 2).

3. Reassessment the day after the suspected SRC (HIA 3).

Although a three-stage process is advised for SRC assessment, riders should be assessed more frequently if they exhibit any suspected concussion signs and/or symptoms and appropriate medical management undertaken.

## HIA (head injury assessment) 1

**Figure 2:**
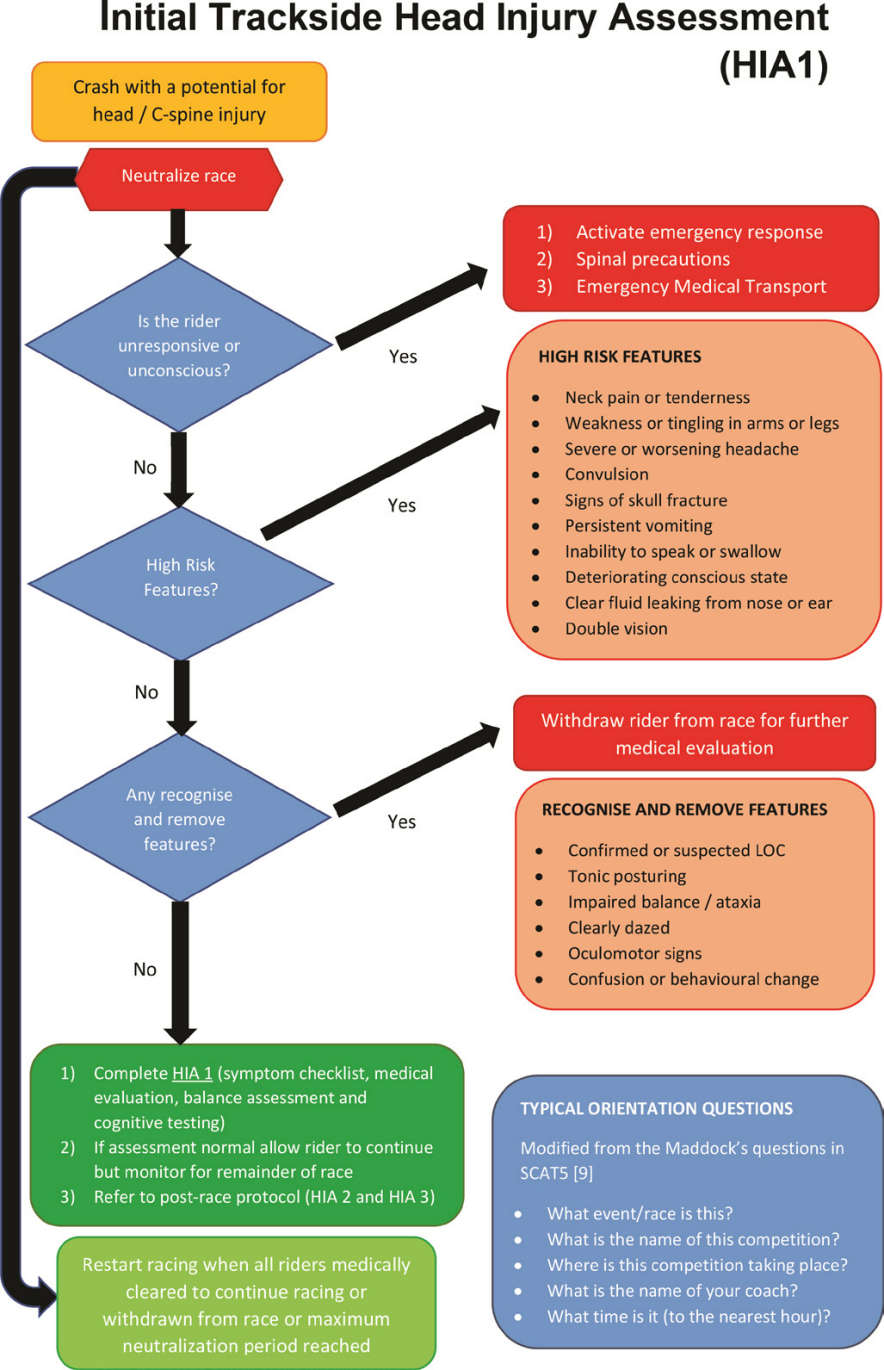
HIA (head injury assessment) 1.

First, riders who sustain a potential SRC injury should be identified by their team staff, team doctors, trackside paramedical staff and/or independent trackside doctors via direct observation of the event or video review. HIA 1 then consists of three components (figure 2):

(A) The rider is assessed for the presence or absence of high risk or recognise and remove features that, if present, warrant immediate and permanent withdrawal from the competition, with appropriate medical management, for example, with neck pain, removal of the rider from the track with full spinal precautions and transported to hospital.

(B) If there are no high risk or recognise and remove features, a uniform trackside screening assessment is completed, including a symptom checklist, medical evaluation, balance assessment and cognitive tests performed by the event doctor, team doctor or independent trackside doctor.

(C) Medical assessment by the race doctor, team doctor or independent trackside doctor.

Those displaying any of the high risk or recognise and remove features will be immediately withdrawn from the competition and not allowed to return to competition that day. Others with the potential for SRC but without any high risk or recognise and remove features, will undergo a uniform medical assessment at the side of the track using the HIA1 protocol (figure 2). The HIA1 assessment will require the race to be neutralised until the assessment is complete and this concussion protocol implementation within track cycling events will, therefore, require collaboration with the Union Cycliste Internationale (UCI), with potential rule changes required.

## HIA (head injury assessment) 2

**Figure 3:**
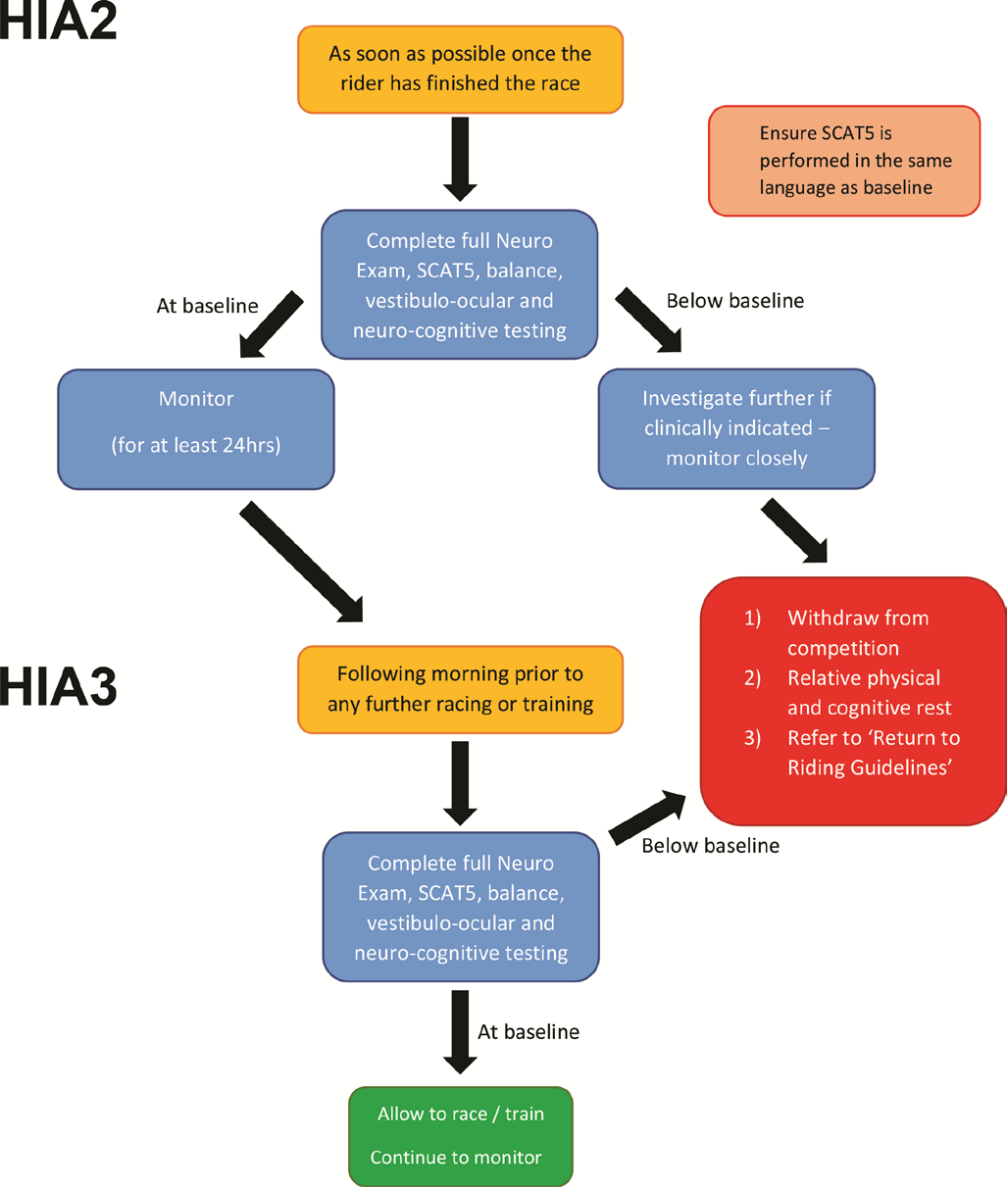
Subsequent head injury assessment (HIA2 and HIA3).

Those who ‘pass’ the HIA 1 assessment will then be able to complete the race as long as they have no further symptoms/signs suggestive of SRC. Following completion of the race, the rider will then undergo a further medical evaluation for SRC (HIA 2). HIA2 consists of a SCAT5,^7^ neurocognitive tools (such as a digital symbol substitution test) and a full neurological exam (figure 3). The HIA2 results should be compared with the rider’s baseline data, which would have been completed at preseason (or precompetition) screening.

## HIA (head injury assessment) 3

The third stage, HIA3, is undertaken after one night’s rest and consists of repeating the clinical tests undertaken within HIA2. HIA3 is used to identify a late diagnosis of SRC.

## How can this HIA protocol be practically implemented into track cycling?

The practical considerations of implementing the SRC protocol into track cycling need to be considered and this will require collaborative work with the UCI and partners. One key point to consider is who should actually perform the in-race HIA 1 assessment, and the number of doctors required trackside to undertake these medical evaluations. Another issue is the time needed for the HIA 1 assessment. Unless the officials stop the race, riders who have crashed must re-join the race before the bunch has completed five laps or else be disqualified. This can leave less than a minute to complete a medical assessment, which is an unrealistic time to complete a concussion assessment within.We, therefore, suggest a fixed time to conduct the proposed head injury assessment, with some sports (eg, rugby) currently allowing 12 min for the medical evaluation.^8^ However, a similar time for SRC assessment in track cycling, through neutralisation of the race (pausing the race while riders are assisted off the track, medically assessed and the track itself is assessed for damage), would have an effect on the race scheduling and with it, riders’ race preparations. This would need to be considered by the event broadcasters and the UCI when scheduling events. An alternative to race neutralisation could be that riders with suspected SRC are permanently withdrawn from the race to allow the concussion assessment to occur and receive a DNF (did not finish) result.

The UCI will also need to ensure consistent application of the concussion policy across the track cycling discipline. We, therefore, encourage the development of an appropriate education programme for riders, management, race and team medical staff in addition to a wider briefing of the media and the viewing public. Non-medical staff can also play a role in SRC assessment, using the Concussion Recognition Tool,^9^ by recognising potential concussive events and symptoms and then ‘holding’ the rider, not allowing them to return to the race until a medical assessment can take place at trackside. Another issue to consider is language requirements at international events and the fact that the injured rider needs to understand the language of the assessing medical staff. Thus, multiple doctors who speak multiple languages to cover the riders’ languages may be required at international events.

Additionally, if this concussion protocol is established within track cycling, the UCI or another appointed body, will need to monitor its application and ensure consistent application of the policy, with the potential for disciplinary action if the assessment process has been abused.

## Summary

Concussion is very much a topical subject within athletes, spectators and sport administrators. We propose the application of a HIA model within track cycling to facilitate better diagnosis and management of SRC within the sport. We recognise that the proposed model is a ‘first step’ in this process, and we encourage discussion with the sporting community, including the UCI, to challenge and further develop this SRC assessment protocol.
