# Correction: Sports-related concussion (SRC) in track cycling: SRC assessment protocol for elite track cycling

**DOI:** 10.1136/bmjsem-2022-001384corr1

**Published:** 2022-10-25

Gomes C, Jones N, Heron N. Sports-related concussion (SRC) in track cycling: SRC assessment protocol for elite track cycling. *BMJ Open Sport Exerc Med* 2022;8:e001384. doi: 10.1136/bmjsem-2022-001384.

The article has been corrected since it was published online. The authors would like to notify that the Maddocks questions in figure 2 are not the correct ones for the track cycling. To support this, they have replaced figures 1 and 2. And a new figure, figure 3 has been included in the published version as well.

**Figure 1:**
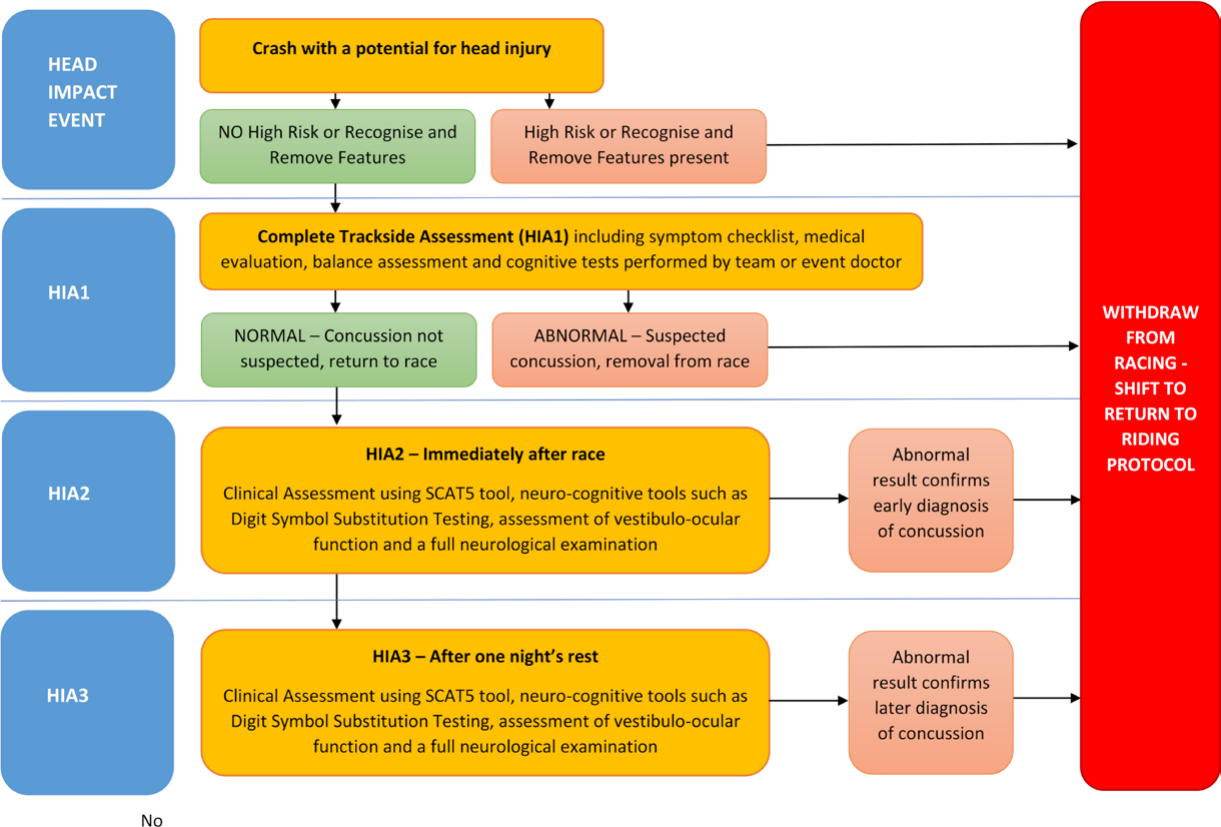
Track Cycling SRC Assessment Protocol.

**Figure 2:**
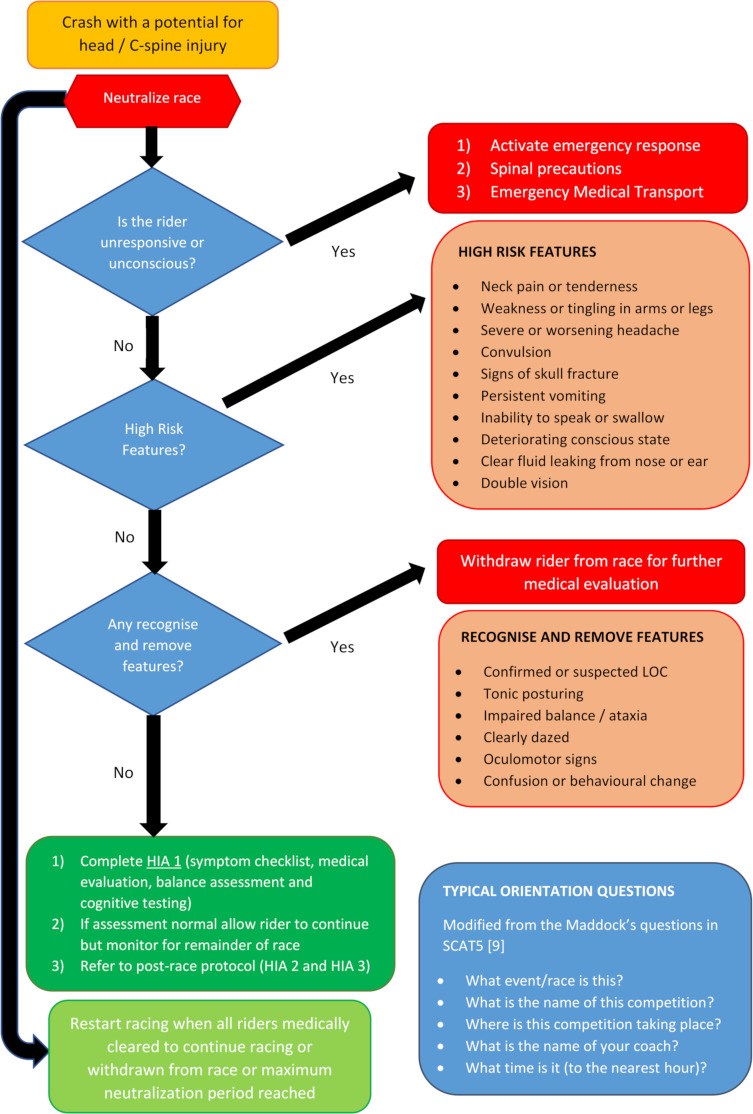
Initial Trackside Head Injury Assessment (HIA1).

**Figure 3:**
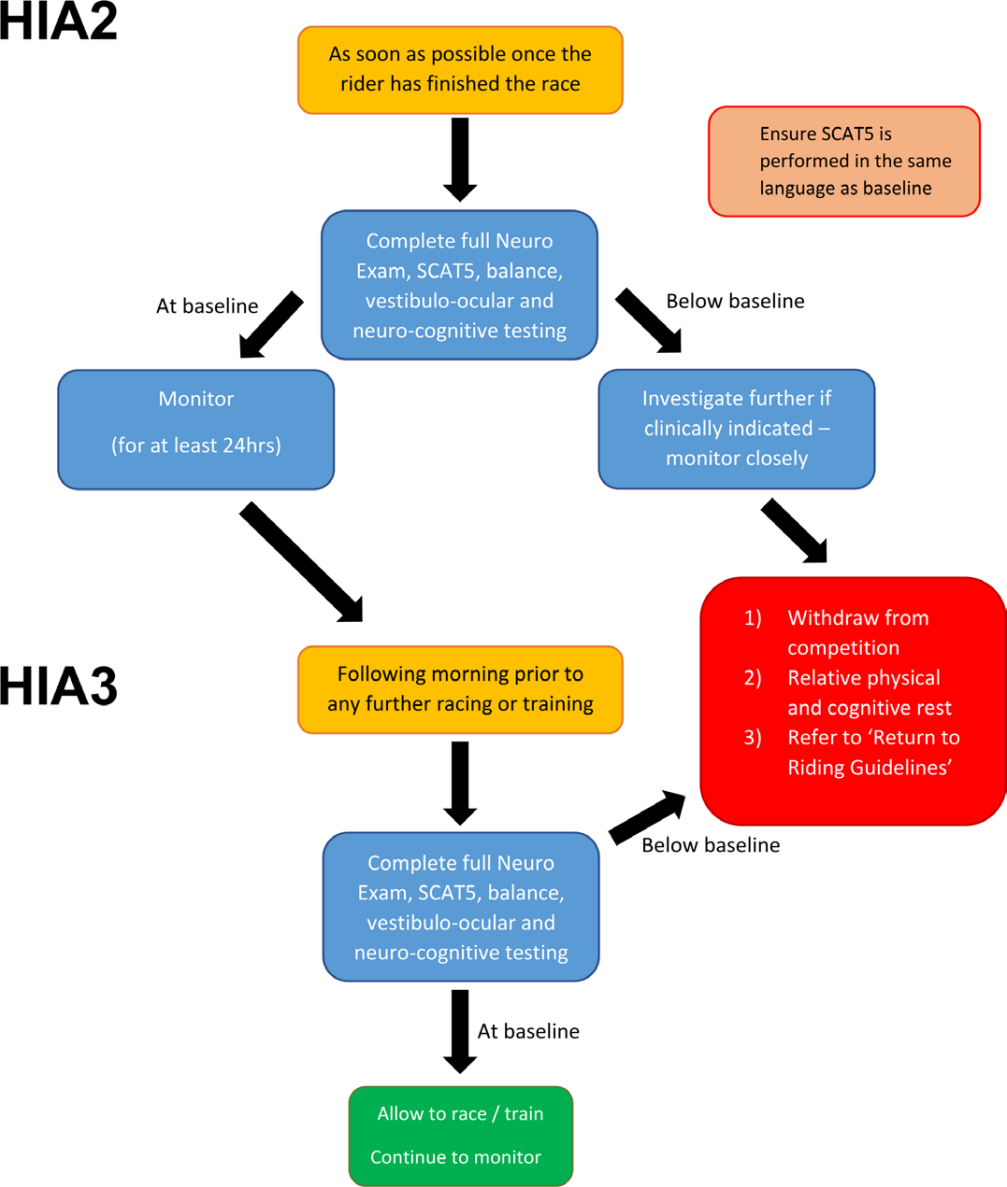
HIA2 and HIA 3 Assessments.

